# Paragangliomas/Pheochromocytomas: Clinically Oriented Genetic Testing

**DOI:** 10.1155/2014/794187

**Published:** 2014-05-12

**Authors:** Rute Martins, Maria João Bugalho

**Affiliations:** ^1^Departamento de Ciências Biomédicas e Medicina, Universidade do Algarve, 8005-139 Faro, Portugal; ^2^Serviço de Endocrinologia, Instituto Português de Oncologia de Lisboa Francisco Gentil E.P.E., 1099-023 Lisboa, Portugal; ^3^Clínica Universitária de Endocrinologia, Faculdade de Ciências Médicas, Universidade Nova de Lisboa, 1169-056 Lisboa, Portugal

## Abstract

Paragangliomas are rare neuroendocrine tumors that arise in the sympathetic or parasympathetic nervous system. Sympathetic paragangliomas are mainly found in the adrenal medulla (designated pheochromocytomas) but may also have a thoracic, abdominal, or pelvic localization. Parasympathetic paragangliomas are generally located at the head or neck. Knowledge concerning the familial forms of paragangliomas has greatly improved in recent years. Additionally to the genes involved in the classical syndromic forms: *VHL* gene (von Hippel-Lindau), *RET* gene (Multiple Endocrine Neoplasia type 2), and *NF1* gene (Neurofibromatosis type 1), 10 novel genes have so far been implicated in the occurrence of paragangliomas/pheochromocytomas: *SDHA, SDHB, SDHC, SDHD, SDHAF2, TMEM127, MAX, EGLN1, HIF2A,* and *KIF1B*. It is currently accepted that about 35% of the paragangliomas cases are due to germline mutations in one of these genes. Furthermore, somatic mutations of *RET, VHL, NF1, MAX, HIF2A,* and *H-RAS* can also be detected. The identification of the mutation responsible for the paraganglioma/pheochromocytoma phenotype in a patient may be crucial in determining the treatment and allowing specific follow-up guidelines, ultimately leading to a better prognosis. Herein, we summarize the most relevant aspects regarding the genetics and clinical aspects of the syndromic and nonsyndromic forms of pheochromocytoma/paraganglioma aiming to provide an algorithm for genetic testing.

## 1. Introduction

Paragangliomas are neuroendocrine tumors that can originate in either the parasympathetic or sympathetic nervous system. Most parasympathetic paragangliomas are chromaffin-negative (meaning that they do not stain brown when exposed to potassium dichromate) and do not secrete catecholamines. Sympathetic paragangliomas (including those derived from the adrenal medulla) are chromaffin-positive tumors that generally secrete catecholamines [1].

The designation of pheochromocytoma appears in the literature associated with different meanings. The World Health Organization (WHO) Tumor Classification defines pheochromocytoma as a paraganglioma derived from the adrenal medulla [2], whilst some authors use the term pheochromocytoma to refer to catecholamine-producing paragangliomas independently of being adrenal or extra-adrenal. In the present revision, we will use the WHO classification.

Sympathetic paraganglia are mainly found in the adrenal medulla but also in the axial regions of the trunk along the prevertebral and paravertebral sympathetic chains and in the connective tissue within/near pelvic organs. In contrast, parasympathetic paraganglia are almost exclusively confined to the head and neck in the vicinity of major arteries and nerves [3]. Paragangliomas can be categorized into functioning/nonfunctioning according to their ability to secrete catecholamines. Sympathetic tumors (including pheochromocytomas) tend to hypersecrete catecholamines (up to 90%), whereas only about 5% of parasympathetic paragangliomas secrete catecholamines [4, 5]. Among the functioning paragangliomas, the pheochromocytomas are the most frequent (80–85% of the cases) followed by the extra-adrenal abdominal paragangliomas [4–6].

The clinical presentation of these patients is highly variable, with most symptoms being nonspecific and mimicking other clinical conditions. Headaches, hypertension, tachycardia, diaphoresis, pallor, anxiety, and panic attacks are the most frequent signs and symptoms at presentation [6]. The classic triad of palpitations, headaches, and profuse sweating altogether can provide a specificity of more than 90% [7]. Paroxysmal hypertension is frequent, either in patients with sustained hypertension or normal blood pressure. In fact, these patients typically present paroxysmal signs and symptoms (lasting less than an hour) that result from episodic release of catecholamines usually due to a triggering factor (surgery, stress, exercise, certain foods, medications, alcohol, etc.) [6]. Signs and symptoms in patients harboring parasympathetic paragangliomas are related to their mass effect causing compression of adjacent tissues and nerves, such as cranial nerves IX–XII [8, 9].

Paragangliomas are rare tumors occurring with an overall estimated incidence of 1/300 000, with an average age at diagnosis of around 40 years and no gender differences [2, 4, 10, 11]. However, the incidence of these tumors is much higher at autopsy (≈0.05%), probably due to the often-asymptomatic clinical course of these tumors that, on the other hand, may result in premature mortality [12–14]. In hypertensive patients' series, the prevalence of paragangliomas/pheochromocytomas ranges from 0.1 to 0.6% [15–17].

Although most tumors are benign, about 10% of pheochromocytomas and 15% to 35% of extra-adrenal paragangliomas are malignant [18]. Prior to the appearance of distant metastases, commonly found in lungs, bone, or liver, there are no reliable histological, genetic, or imaging markers to predict malignancy of these tumors [18]. The histological PASS (Pheochromocytoma of the Adrenal gland Scaled Score) system was developed to predict the risk of malignant pheochromocytomas; however, the high interobserver and intraobserver variations make this score of limited clinical use [19–21]. Some studies have also pointed out that the size and location of the tumor, the downregulation of metastasis suppressor genes, early onset postoperative hypertension, high levels of plasma/urine metanephrines, immunochemical expression of the angiogenesis-related genes, and high levels of serum chromogranin A at the time of diagnosis, amongst many others, increase the likelihood of malignant pheochromocytoma [18, 22–25]. Of particular importance are the germline mutations in the* SHDB* gene (discussed in detail later), which have been associated with up to 72% of malignant tumors [26].

Paragangliomas can be classified into either sporadic or familial. In the last years, our knowledge concerning the familial forms of paragangliomas has greatly improved. Additionally to the genes involved in the classical syndromic forms:* VHL* gene in von Hippel-Lindau disease,* RET* gene in Multiple Endocrine Neoplasia type 2 (MEN 2), and* NF1* gene Neurofibromatosis type 1, 10 novel genes have so far shown to be implicated in the occurrence of paragangliomas/pheochromocytomas [27–29]. Amongst these, the most relevant are those of the mitochondrial succinate dehydrogenase (SDH) complex subunits genes (*SDHA*,* SDHB*,* SDHC,* and* SDHD*) and one complex cofactor,* SDHAF2*, mainly involved in head and neck and abdominal paragangliomas and initially discovered by Baysal et al. [30–34]. More recently, the* TMEM127*,* MAX*,* HIF2A*,* EGLN1*,* KIF1B, *and* H-RAS* complete the list of susceptibility genes implicated in the development of paragangliomas/pheochromocytomas [35–40]. So far,* H-RAS* mutations have been identified only at a somatic level.

Pheochromocytoma (here meaning catecholamine-secreting paraganglioma) was known as the 10% tumor, meaning that 10% of cases were familial, 10% bilateral, 10% malignant, and 10% extra-adrenal [1]. The 10 percent dogma concerning the hereditary forms of these tumors was completely discarded by a study in 2002 by Neumann et al. [41]. In this study, it was found that 24% of the patients who presented with nonsyndromic pheochromocytoma and without family history of the disease had mutations in* VHL*,* RET*,* SDHD,* and* SDHB* genes. Younger age at presentation (24.9* versus* 43.9 years of age), multiple tumors (32%* versus* 2%), and presence of extra-adrenal tumors (28%* versus* 8%) were significantly associated with the presence of a mutation [41]. In 2006, a study comprising a larger number of patients with pheochromocytoma/paraganglioma showed that 33% of the patients carried germline mutations in one of the following genes:* VHL*,* RET*,* NF1*,* SDHB,* and* SDHD* [42]. So, it is currently accepted that up to 35% of paragangliomas/pheochromocytomas are associated with an inherited mutation [43, 44].

In this review, we summarize the clinical and genetic aspects of the syndromic and nonsyndromic forms of pheochromocytoma/paraganglioma. The risk of developing pheochromocytoma/paraganglioma will be addressed for each gene. A clinically oriented strategy for genetic testing will be discussed.

## 2. Genetics of Paragangliomas/Pheochromocytomas

### 2.1. Syndromic Forms

#### 2.1.1. von Hippel-Lindau

von Hippel-Lindau (VHL) disease is an autosomal dominant syndrome characterized by a variety of benign and malignant tumors including retinal and central nervous system hemangioblastomas, clear renal cell carcinoma and renal cysts, pheochromocytomas, pancreatic islet cell tumors and pancreatic cysts, epididymal cystadenomas, and endolymphatic sac tumors [27].

This disease affects about 1 in 36 000 live births and is divided into 2 clinical categories according to absence (type 1) or presence (type 2) of pheochromocytomas, respectively [45, 46]. VHL type 2 is further divided in type 2A, identifying patients with low risk of developing clear renal cell carcinoma, type 2B, for patients with high risk of developing clear renal cell carcinoma, and type 2C, for patients that only present pheochromocytomas without the other classical lesions of VHL disease [27]. Pheochromocytomas occur in 10–20% of VHL patients, typically around 30 years, but rare cases have been described below the age of 10. About 5% of pheochromocytomas in VHL disease are malignant [27, 47, 48]. Due to the early onset of these tumors and frequent absence of signs and symptoms, it has been proposed that catecholamine screening should begin at the age of 2, especially in patients with a familial history of pheochromocytomas [47]. VHL-associated pheochromocytomas secrete mostly norepinephrine due to low or absent expression of phenylethanolamine N-methyltransferase; thus, patients present with increased plasma and urinary normetanephrine [49]. The adrenal medulla is the most common paraganglia affected in VHL type 2 patients but rare sympathetic and parasympathetic paragangliomas have also been described [27, 47, 50]. Pheochromocytomas are often bilateral and generally have a good prognosis [51, 52].

von Hippel-Lindau protein (pVHL) is a tumor suppressor protein that regulates the activity of hypoxia-inducible factor alpha (HIF*α*) and several other proteins involved in tumorigenesis [53]. In normoxic conditions, pVHL binds to the *α* subunits of HIF1 and 2, targeting it for ubiquitination and proteasomal degradation. Conversely, in hypoxic conditions or when* VHL* gene is mutated, HIF*α* is able to interact with HIF*β*, inducing the transcription of hypoxia-inducible genes, leading to an increased expression of angiogenic growth and mitogenic factors [53–55]. This disruption of pVHL-mediated degradation of HIF will ultimately contribute to tumor formation through multiple mechanisms [53].

*VHL* gene was mapped to the short arm chromosome 3 (3p25), it comprises 3 exons that encode for the 2 isoforms of the pVHL protein [56]. More than 150* VHL* germline mutations have been associated to the VHL disease. These mutations are missense, deletion, nonsense, or frameshift mutations and are distributed throughout the coding sequence [57, 58]. Although genetic testing studies have been able to identify mutations in virtually every VHL-affected family, diagnosis is still challenging in up to 20% of affected kindreds in which a* de novo* mutation occurs [58, 59]. Genotype-phenotype correlation studies have shown that VHL type 1 families frequently harbor* VHL* deletions or nonsense mutations, whereas families at risk for developing pheochromocytoma (type 2 families) almost invariably present with* VHL* missense mutations [57, 58, 60]. Particularly, missense mutations at codon 167 were associated with a high risk of developing pheochromocytoma (53% and 82% at ages 30 and 50 years, resp.) [60].* VHL* mutations associated with the phenotype 2A or 2B have been shown to affect the proteasomal degradation of HIF1, whereas type 2C mutations do not disrupt the ability of pVHL to downregulate HIF1, suggesting that pheochromocytoma formation is not related with HIF1 expression levels [61, 62]. It has been proposed that VHL-associated pheochromocytoma tumorigenesis is related with an abnormal extracellular matrix formation and to upregulation of tyrosine hydroxylase, leading to increased catecholamine synthesis [61, 63, 64].

#### 2.1.2. Multiple Endocrine Neoplasia Type 2

Multiple endocrine neoplasia type 2 (MEN 2) is an autosomal dominant cancer syndrome characterized by the association of medullary thyroid carcinoma (MTC), pheochromocytoma, and hyperparathyroidism [29]. Depending on the most frequent manifestations, there are three subtypes MEN 2A, MEN 2B, and Familial MTC (FMTC): in MEN 2A patients, MTC is present in virtually all patients, unilateral or bilateral pheochromocytoma in 50% of cases, and multigland parathyroid tumors in 20–30% of cases; in MEN 2B patients, the third component (hyperparathyroidism) is not present, MTC has an earlier onset, and there are developmental alterations such as multiple mucosal ganglioneuromas and a “marfanoid” habitus; in FMTC patients, MTC is the single manifestation [65–67]. MEN 2A is the most frequent subtype representing over 75% of MEN 2 cases [66]. It is now accepted that FMTC might be a variant of MEN 2A with a lower clinical penetrance of pheochromocytoma [67, 68].

The genetic basis for MEN 2 syndrome lies within the long arm of chromosome 10 (10q11.2), where the* RET* (REarranged during Transfection) protooncogene is located. It comprises 21 exons that encode for a tyrosine transmembrane receptor with three domains: extracellular, transmembrane, and intracellular. When a ligand of the glial-derived neurotropic factor (GDNF) family binds to RET protein, it triggers RET dimerization and autophosphorylation, inducing a signaling phosphatidylinositol 3′-kinase- (PI3K-) mediated cascade that regulates cell proliferation and apoptosis. This requires the presence coreceptors of the GDNF family receptor-*α*1–4 (GFR-*α*1–4) at the cell surface [69].

MEN 2 subtypes have been associated with specific* RET* mutations. More than 98% of MEN 2A families present with missense mutations in one of five codons: 609, 611, 618, 620 (exon 10), or 634 (exon 11). Codon 634 mutations represent almost 90% of MEN 2A cases and a cysteine to arginine substitution at this codon (p.Cys634Arg) is found in more than 50% of cases [70–73]. All these mutations affect cysteine residues in the RET extracellular domain and induce a ligand-independent dimerization of RET, leading to a constitutive activation of its intrinsic tyrosine kinase [74–76]. About 80% of patients with FMTC present with a similar mutational spectrum of MEN 2A, but mutations are relatively evenly distributed among codons 618, 620, and 634 [70–73]. Interestingly, the p.Cys634Arg mutation is almost never found in FMTC families [67]. Generally, MEN 2B tumors are a consequence of mutations in the substrate binding pocket of the RET tyrosine kinase. A single missense mutation in codon 918 (p.Met918Thr) is responsible for over 90% of MEN 2B cases, whereas other rare mutations have been described in exons 14 and 15 [69, 71–73]. The American Thyroid Association (ATA) proposed the categorization of patients into four risk levels (A to D) based on the mutation identified and on the genotype-phenotype correlation. Clinical recommendations concerning prophylactic surgeries in asymptomatic individuals depend on the attributed risk level [67].

Pheochromocytomas in MEN 2A and B syndromes are generally benign tumors and bilateral in >50% of the patients [29]. Extra-adrenal paragangliomas have been described but are very rare [29, 77]. The biochemical phenotype of these tumors is increased plasma and urinary levels of metanephrine as a result of epinephrine hypersecretion, possibly due to overexpression of phenylethanolamine N-methyltransferase [49]. Large cohort series show that malignancy affects less than 5% of MEN 2-associated pheochromocytomas [78, 79].

Based on a large study enrolling 323 MEN 2A patients, Quayle et al. reported an overall penetrance of pheochromocytoma of 32%, with a median age at diagnosis of 34 years; the earliest pheochromocytoma was observed at 15 years; bilateral pheochromocytomas were observed in 66% of patients; the following codon-specific expression of pheochromocytoma was observed: codon 634 was expressed in 50%, codon 618 was expressed in 22%, codon 620 was expressed in 9%, and codon 609 was expressed in 4%. The mean age at diagnosis did not differ amongst these codon-grouped patients [80].

Childhood pheochromocytoma is rare in MEN 2, but reports at 12 years of age have occurred for both the 918 and 634* RET* mutations [79, 81]. Therefore, the ATA recommends that pheochromocytoma screening (by plasma or 24-hour urine fractionated metanephrines) should begin by age 8 in carriers of* RET* mutations associated with MEN 2B and mutated* RET *codons 634 and 630 and by the age 20 years in carriers of other MEN 2A* RET *mutations. Patients with* RET* mutations associated only with FMTC should be screened at least periodically from the age of 20 years [67].

#### 2.1.3. Neurofibromatosis Type 1

Neurofibromatosis type 1 (NF 1), or von Recklinghausen's disease, is an autosomal dominant disorder clinically diagnosed by six or more* cafe au lait* macules; two or more cutaneous/subcutaneous neurofibromas or a single plexiform neurofibroma; axillary or inguinal freckling; optic nerve glioma; two or more Lisch nodules (iris hamartomas); dysplasia of long bones or pseudarthrosis; and a first degree relative with NF1 [28, 82]. Patients with NF 1 are also at higher risk than general population of developing various tumors such as peripheral nerve sheath tumors, gastrointestinal stromal tumors, rhabdomyosarcoma, breast cancer, and pheochromocytomas [83, 84]. Worldwide birth incidence of NF 1 is 1 in 2 500–3 000 and prevalence is at least 1 in 4 000 [84].

NF 1 is caused by loss of function mutations in the tumor-suppressor* NF1* gene [85]. This gene is located on chromosome 17q11.2 comprising 60 exons that encode for neurofibromin, a negative regulator of RAS proteins. Neurofibromin is a GTPase activating protein that promotes the conversion of active RAS-GTP to its inactive form, RAS-GDP. Mutations in* NF1* gene result in constitutive activation of RAS activity triggering a kinase cascade and the activation of mitogen-activated protein kinases (MAPK), mammalian target of rapamycin (mTOR), and PI3 K pathways, therefore regulating the transcription of genes associated with cell proliferation, cell death, differentiation, and migration [86]. Mutational analysis in NF 1 patients remains a considerable challenge due to the occurrence of different types of mutations (nonsense, missense, or deletions) that span the entire length of the* NF1* gene, the presence of 36 pseudogenes, and the fact that nearly half of NF 1 cases present* de novo* mutations [87, 88].

Pheochromocytomas are a rare feature in NF 1, affecting approximately 0.1% to 6% of all patients [83, 89]. A prevalence rate as high as 13% has been reported in autopsy series, suggesting that the diagnosis of pheochromocytoma may be missed in some NF 1 patients [89]. The mean age at presentation of pheochromocytoma is 42 years. The majority of patients have unilateral adrenal tumors, whereas 10% of patients present with bilateral and 6% abdominal tumors. Malignant pheochromocytomas were identified in 12% of the NF 1 patients [42, 89]. Similarly to MEN 2-associated pheochromocytomas, in NF 1 these tumors have been shown to produce more epinephrine and less norepinephrine, resulting in increased levels of metanephrine [49]. Although pheochromocytoma NF 1-associated is rare, due to the risk of malignancy, it has been proposed that any patient with hypertension/paroxysmal hypertension or with symptoms of catecholamine excess, such as headache, sweating, palpitations, or anxiety, should undergo measurement of 24-hour urine or plasma metanephrines [28].

Unlike mutations in VHL or MEN 2 disorders, NF 1 mutations that offer an increased risk in pheochromocytoma remain to be identified. A study carried out by Bausch et al. in NF 1 patients with associated pheochromocytoma showed that the cysteine-serine rich domain was affected in 35% of the cases whereas the Ras GTPase activating protein domain in only 13%, suggesting that the cysteine-serine rich could play a role in the formation of NF1-associated pheochromocytoma. Moreover, in accordance with the Knudson's two-hit theory that states that pheochromocytoma development requires biallelic inactivation, loss of heterozygosity (LOH) was shown in NF 1-related pheochromocytoma. No association was found between* NF1* mutational genotype and the clinical features of pheochromocytoma [90].

### 2.2. Familial Paraganglioma Syndromes (*SDHx* and* SDHAF2*)

Familial paraganglioma syndromes (PGLs) are a group of autosomal dominant disorders responsible for the development of paragangliomas/pheochromocytomas caused by mutations in the genes encoding for the succinate dehydrogenase (SDH) mitochondrial complex. SDH or respiratory complex II is an enzyme complex that catalyses the oxidation of succinate to fumarate in the Krebs cycle and participates in the electron transport chain [91]. SDH is composed of 4 subunits encoded by the corresponding genes:* SDHA*,* SDHB*,* SDHC,* and* SDHD*. Complex subunits A (flavoprotein) and B (iron-sulfur protein) constitute the catalytic core of the enzyme, while subunits C and D anchor the complex to the inner mitochondrial membrane. In general, inactivating mutations in one of the* SDHx* genes leads to accumulation of succinate and formation of reactive oxygen species, stabilizing HIF*α* and activating hypoxia-dependent pathways [91]. Four PGL syndromes have been described: types 1, 2, 3, and 4, caused by mutations in the* SDHD*,* SDHAF2* (responsible for the flavination of subunit A),* SDHC,* and* SDHB*, respectively [30–33]. Immunohistochemistry can be used to triage genetic testing of paraganglioma/pheochromocytoma. Particularly for SDHB immunohistochemistry, a negative staining is more commonly found associated with* SDHB* mutation, whereas a weak diffuse staining often occurs with* SDHD* mutation [92, 93]. Functioning* SDHx* paragangliomas sometimes release dopamine and/or norepinephrine, originating raised plasma levels of methoxytyramine, contributing to distinguish* SDHx* patients from those with* VHL*,* RET,* or* NF1* mutations [49, 94]. However, methoxytyramine should be regarded as a useful biomarker of malignancy in the setting of paraganglioma/pheochromocytoma independent of the underlying gene. Penetrance and clinical presentation of PGL syndromes varies significantly with the underlying mutation [95].

#### 2.2.1. PGL 1 Syndrome

PGL 1 syndrome is caused by mutations in* SDHD* gene, which are inherited in an autosomal dominant fashion with a predominant paternal transmission, suggesting a maternal imprinting of this gene [30, 34, 44, 96]. However, rare cases of maternal transmission have been described and the precise mechanism responsible for this parent-of-origin effect remains to be elucidated [97–99]. A three-hit model has been hypothesized requiring a* SDHD* mutation, loss or mutation of the wild-type* SDHD* allele, and loss of a further imprinted (paternally silenced and maternally active) tumor suppressor gene from chromosome 11 [99, 100]. PGL 1 patients generally present with multiple benign parasympathetic head and neck paragangliomas, but multiple sympathetic and adrenal tumors are also very frequent. In fact, Neumann et al. have shown that among 34 patients with mutations in* SDHD* gene, 79% had head and neck paraganglioma, 53% had pheochromocytoma, and 39% thoracic/abdominal paraganglioma, whereas 74% of the patients presented with multiple tumors [96]. Mean age at presentation is around 30 years [43, 96, 101, 102]. Ricketts et al. estimated the risk of developing head and neck paragangliomas at 71% and the risk of pheochromocytoma at 29%, at age 60 [102]. Malignancy has rarely been found in* SDHD*-derived sympathetic or parasympathetic paragangliomas [43, 96, 101–105]. Several different mutations have been described in exons 2–4 of* SDHD*, mainly nonsense, missense, and frameshift, but its relation with the phenotypic expression of the disease is still unclear [43, 96, 101, 102].

#### 2.2.2. PGL 2 Syndrome

Familial PGL 2 syndrome is a very rare condition characterized by multiple head and neck paragangliomas, of which only few cases have been reported [106, 107]. It happens as a consequence of mutations in* SDHAF2* gene (also known as* SDH5*) that encodes for a succinate dehydrogenase complex assembly factor 2 (SDHAF2), which is responsible for the flavination of SDHA enabling SDH complex activity [31]. To our knowledge, only two apparently unrelated kindreds (of Dutch and Spain origin) have been described as carriers of a missense mutation in this gene, c.232G > A (p.Gly78Arg) [31, 106, 107]. Both kindreds show a high penetrance for this mutation, which has a paternal mode of transmission. Among the 16 mutations carriers of the largest branch of the Dutch family, considered as at-risk patients, 11 patients had head and neck tumors, out of which 10 had multiple tumors (91%). The mean age of diagnosis was 33 years [107].

The scarcity of* SDHAF2* mutations was reinforced by the failure to document mutations in this gene among 315 patients with paraganglioma and without mutations in the* SDHD*,* SDHC,* or* SDHB* genes. Nonetheless, it is justified to screen for* SDHAF2* mutations in young patients with isolated head and neck paragangliomas or in individuals with familial antecedents who are negative for other risk genes [106].

#### 2.2.3. PGL 3 Syndrome

Mutations in the* SDHC* gene are causative for familial PGL syndrome 3, which has an autosomal dominant mode of transmission without a parent-of-origin effect [32]. This is a rare condition characterized by benign parasympathetic head and neck tumors, but rare cases of sympathetic paragangliomas and pheochromocytomas have been described [44, 108–111]. In the studies by Burnichon et al. [44] and Schiavi et al. [112], the mean age at presentation was 38 (17–70) and 46 years (13–73), respectively.

About 4% of paraganglioma patients carry mutations in the* SDHC* gene [44, 112]. Different types of mutations (missense, nonsense, splicing, deletions, and insertions) encompassing the whole* SDHC* gene have been found [44, 105, 109, 112]. Malignancy associated with* SDHC* gene is extremely rare with only two cases described so far, with distinct causal mutations [113, 114].

#### 2.2.4. PGL 4 Syndrome

Familial PGL 4 syndrome is characterized by abdominal and pelvic catecholamine-secreting paragangliomas, which can also be present in adrenal medulla and head and neck [94, 102, 112, 115]. Symptoms are those classically associated with paraganglioma/pheochromocytoma (headache, palpitations, and diaphoresis) but can also be due to a mass effect rather than catecholamine secretion [94]. Mean age at diagnosis is around 32 years [94, 96, 102, 115]. Primary tumors are usually large and associated with a high rate of malignancy ranging from 31 to 72% of patients [26, 94, 96, 115].

Germline mutations in* SDHB* gene, which encodes for the iron sulfur subunit of the SDH complex (subunit B), are responsible for PGL 4 familial syndrome [33]. Functional assays have shown that these mutations lead to stabilization of HIF1*α*, causing overexpression of hypoxia-induced angiogenic pathway genes, such as VEGF (vascular endothelial growth factor) and EPAS1 (endothelial PAS domain protein 1), providing therefore support for tumor growth [116–118]. Loss of heterozygosity has been shown to occur as a consequence of* SDHB *mutations [33, 116]. Of interest, mutations in* SDHB* gene have also been associated with an increased susceptibility to develop other neoplasms, namely, renal cell carcinoma, gastrointestinal stromal tumors, papillary thyroid cancer, and neuroblastoma [96, 102, 115, 119].

A wide spectrum of* SDHB* mutations have been found associated with PGL 4, namely, missense, frameshift, splicing, nonsense, and large deletions. However, several studies have failed to unveil genotype-phenotype correlations, particularly in what concerns tumor location, age of presentation, and aggressiveness of the tumor [94, 120]. Since mutations in* SDHB* gene are the most frequent cause of metastatic paraganglioma tumors, it has consistently been proposed that all patients presenting with malignant paraganglioma/pheochromocytoma should be tested for* SDHB* gene mutations.

#### 2.2.5. *SDHA*

The long-sought link between* SDHA* gene and paraganglioma development was only unveiled in 2010, when a patient with an extra-adrenal paraganglioma was found to have an* SDHA* missense mutation [34]. Functional studies show that* SDHA*, like other* SDHx* genes, operates as tumor suppressor gene and activates the pseudohypoxic pathway leading to tumorigenesis. Furthermore, in accordance with Knudson's two-hit hypothesis, it was shown that the* SDHA*-mutated tumors have lost the wild type allele [34, 121]. The few* SDHA*-affected individuals described so far have presented with distinct phenotypic characteristics: pheochromocytoma, sympathetic (abdominal and thoracic), and parasympathetic head and neck paragangliomas [34, 121–123]. The reported age at diagnosis is highly variable. Missense and nonsense mutations have been found, without any genotype-phenotype correlation [34, 121–123]. Recently,* SDHA* gene mutations have also been implicated in the development of gastrointestinal stromal tumors [124, 125].

### 2.3. Other Susceptibility Genes

#### 2.3.1. *TMEM127*

*TMEM127* is a tumor suppressor gene initially identified as a pheochromocytoma susceptibility gene [37] and later also associated with the development of paragangliomas of head and neck and extra-adrenal abdominal paragangliomas [126–130].* TMEM127* gene encodes a highly conserved transmembrane protein, transmembrane protein 127, which is associated with several cellular organelles and thought to limit mTORC1 activation thus controlling protein synthesis and cell survival [37]. Mutations in this gene are inherited in an autosomal dominant fashion and induce tumor development by enhancing the kinase-dependent signaling pathways, similarly to mutated* RET* and* NF1* genes [37]. Patients may present either unilateral or bilateral pheochromocytomas. The mean age at diagnosis is around 42 years and the risk of malignancy is very low (≈1%). The prevalence of* TMEM127* mutations in patients with paraganglioma/pheochromocytoma varies between 0.9 and 2%. Different missense, frameshift, or nonsense* TMEM127* mutations have been found across the three exons of the gene [126, 129].

#### 2.3.2. *MAX*

Comino-Méndez et al. identified mutations in* MAX* gene as responsible for the development of bilateral pheochromocytoma in eight index patients [38]. This association was further confirmed by another study comprising 1,694 patients with paraganglioma/pheochromocytoma [131]. The latter study documented* MAX* germline mutations in 23 nonrelated patients, all with adrenal tumors; among the 19 patients considered for phenotypic associations, 13 (68%) presented with bilateral or multifocal pheochromocytoma and 16% developed additional thoracoabdominal paragangliomas [131]. Median age at diagnosis was 34 years and 37% of the patients had familial antecedents. Overall,* MAX* germline mutations were found in 1.12% of patients without other mutations [131]. Both studies presented patients with metastatic disease, but further research is required to ascertain the risk of malignancy associated with* MAX* mutations [38, 131].* MAX* tumors have an intermediate biochemical phenotype with a predominant normetanephrine release [131, 132].

*MAX* (myc-associated factor X) gene is a tumor suppressor gene that encodes for MAX protein, which is a component of the MYC-MAX-MXD1 complex that regulates cell proliferation, differentiation, and apoptosis [38, 133]. Mutations in* MAX* gene have a paternal mode of transmission and are responsible for the loss of the wild type allele with consequent abrogation of protein expression. Consequently, inhibition of MYC-dependent cell transformation by MAX protein is disrupted, causing tumor development [38].

#### 2.3.3. *HIF2A* and* EGLN1*

As stated before in the context of VHL disease, HIF*α* proteins (HIF1*α*, HIF2*α*, and HIF3*α*) are transcription factors that respond to oxygen concentrations in tissues. Under hypoxic conditions, stabilization of HIF*α* proteins occurs, allowing transcription of genes involved in angiogenesis, glycolysis, erythropoiesis, apoptosis, proliferation and growth [134]. Mutations in* VHL* and* SDHx* genes have been shown to induce pseudohypoxic states that induce the development of paragangliomas/pheochromocytomas. In 2012, Zhuang et al. described two somatic mutations in the gene encoding of the hypoxia-inducible factor 2*α* (*HIF2A*) in two patients with polycythemia and multiple paragangliomas (one of the patients also presented with somatostatinomas). Functional assays show that both mutations affected pVHL hydroxylation, impairing HIF2*α* degradation leading to an intact/increased transcriptional activity of genes downstream of HIF2*α*, such as* VEGFA* and erythropoietin [39]. These findings were further corroborated by other recent studies that confirmed somatic* HIF2A* gain-of-function mutations as causative for the development of polycythemia and multiple paragangliomas/pheochromocytomas and somatostatinomas in patients, corresponding to a novel syndrome [135–142]. The occurrence of multiple tumors presenting the same somatic mutations without familial history suggests the occurrence of a* de novo *postzygotic event early in the embryogenesis [39, 138]. Somatic mutations in* HIF2A* have also been identified in sporadic pheochromocytomas/paragangliomas in the absence of erythrocytosis [137].

EGLN (egg-laying-defective nine) family of proteins (also called PHD or HPH) are responsible for hydroxylation of prolyl residues of HIF*α* under normoxic conditions, allowing pVHL binding and proteosomal degradation of HIF*α* proteins [134]. The association between EGLN proteins and paraganglioma development was first established by Ladroue et al., by reporting a patient presenting with erythrocytosis and recurrent abdominal paragangliomas who carried a germline mutation in the* EGLN1* gene (formerly known as* PHD2*) [35]. Loss of heterozygosity involving the tumor wild type* EGLN1* allele suggests that* EGLN1* may act as tumor-suppressor gene. Functional studies indicate stabilization of HIF2*α* in the presence of EGLN1 mutant protein [35]. Additional research is required to disclose the role of* EGLN1* mutations in paragangliomas.

#### 2.3.4. *H-RAS*

The RAS-ERK pathway has long been associated with the development of cancer [143]. Regarding paragangliomas/pheochromocytomas, it is currently accepted that there are 2 distinct tumorigenesis clusters according to their transcriptional profile: a pseudohypoxic cluster (associated with mutations in* VHL/SDHx/EGLN1* genes) and a kinase receptor-signaling cluster (associated with* RET/NF1/TMEM127/MAX/KIF1B* mutations) [144]. Evidence for a novel link between the latter cluster and paraganglioma development has been provided by Crona et al., through the identification of somatic mutations in* H-RAS* gene in four male patients presenting with pheochromocytoma (3 patients) and paraganglioma (1 patient) [40]. Very recently, the same authors have described an additional* H-RAS* somatic mutation in a patient with unilateral pheochromocytoma [145].

#### 2.3.5. *KIF1B*

Kinesin family member 1B (*KIF1B*) gene expression results in two protein isoforms, KIF1B*α* and KIF1B*β*, which are motor proteins involved in the anterograde transport of mitochondria and synaptic vesicle precursors, respectively [146, 147]. Schlisio et al. firstly associated two* KIF1B *missense mutations as causative of pheochromocytoma in two tumor samples [36]. It was also shown that KIF1B*β* acts downstream from oxygen-dependent prolyl hidroxylase EGLN3 (or PHD3) to induce apoptosis. These loss of function mutations in KIF1B*β* could therefore protect neuroblasts from apoptosis, leading to tumor development [36]. This study was further extended to five relatives of a patient harboring a germline* KIF1B* mutation. These individuals presented unilateral or bilateral pheochromocytoma and other nonneural crest-derived malignancies, such as ganglioneuroma, leiomyosarcoma, and lung adenocarcinoma [148]. Transcriptional analysis of KIF1B*β* mutant pheochromocytomas showed that these tumors are transcriptionally related to* RET* and* NF1*-associated tumors.

## 3. Genetic Testing Strategy in Paraganglioma/Pheochromocytoma

According to the general recommendation for genetic screening of the American Society of Clinical Oncology, all patients with a risk of at least 10% of carrying a genetic mutation should be offered genetic testing [149]. It is currently accepted throughout the literature that about 35% of paraganglioma/pheochromocytoma cases are due to germline mutations in one of the formerly described genes [41–43, 150, 151]. Therefore, it has been proposed by several authors that genetic testing should be performed to all paraganglioma/pheochromocytoma patients [6, 41, 96, 151, 152].

In the clinical setting, hereditary paraganglioma/pheochromocytoma syndromes should be considered in all individuals with paragangliomas and/or pheochromocytomas, particularly those with tumors that are multiple and recurrent and have an early onset (age < 45 years). Absence of a known family history is not enough to exclude this hypothesis. However, there are subgroups of patients with a very low risk as it is the case for those with apparently sporadic paraganglioma/pheochromocytoma after age 50 [65, 151].

There are several meaningful motives in favor of offering the genetic testing to paraganglioma patients. The inherited syndromic forms caused by mutations in* VHL*,* RET,* and* NF1* genes are associated with other malignant tumors. Thus, an early diagnosis of patients will allow an improved lifelong surveillance and forehand treatment with a consequent improved prognosis [27–29]. On the other hand, patients with germline mutations are more likely to have multiple and recurrent paragangliomas/pheochromocytomas, which requires a more close follow-up [6]. Molecular testing of relatives will clarify their genetic status allowing excluding those who did not inherit the mutation from unnecessary and costly diagnostic procedures.

**Figure 1:**
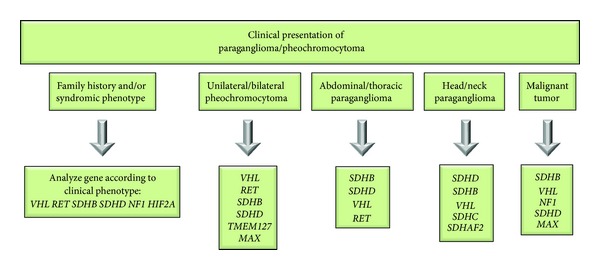
Proposed algorithm for molecular genetic testing of paraganglioma/pheochromocytoma patients. The genes depicted in the boxes are most likely to account for the clinical phenotype and should be analyzed in the proposed order. Mutations in* TMEM127*,* MAX*,* HIF2A,* and* SDHAF2* are extremely rare, so they should only be analyzed when patients are negative for the other gene mutations.

Advanced techniques like whole genome sequencing or next-generation sequencing appear to be promising genetic strategies for testing paraganglioma patients [153–155]. Nevertheless, these techniques are still unavailable for many genetic laboratories or remain far from being cost-effective. To overcome a time consuming gene-after-gene analysis and the difficulties associated with the overlapping clinical features of several syndromic and sporadic forms, different algorithms have been proposed based on a sequential approach and taking into account the patient's family history and clinical presentation. Particular aspects such as the localization of the primary tumor, the biochemical profile, and age at diagnosis are considered of extreme relevance to orient the genetic study. Herein, we propose an algorithm (Figure 1) for genetic testing of paraganglioma/pheochromocytoma patients that incorporates clinical data as well as information derived from previous analytical reviews [48, 65, 150, 151, 156].

Patients presenting specific syndromic features or a positive familial history should be considered for the analysis of the specific genes:* VHL*,* RET*,* SDHB*,* SDHD*,* NF1,* or* HIF2A *[27–30, 33, 39]. For instance, the presence of hemangioblastomas (suggestive of von Hippel-Lindau) or medullary thyroid carcinoma along with pheochromocytoma (suggestive of MEN 2A) strongly implies mutations in* VHL* or* RET* gene, respectively [27, 29]. The coexistence of pheochromocytoma with interscapular pruritic lesions strongly suggests a mutation in codon 634 of the* RET* gene [157]. Expression of disease associated with a paternal transmission mode, consistent with maternal imprinting, orients genetic testing towards specific genes such as* SDHD* or, more rarely,* SDHAF2 *[30, 106]. Adrenal pheochromocytomas (unilateral or bilateral) are more frequently associated with* VHL* and* RET. *Thus,* SDHB*,* SDHD*,* TMEM127,* or* MAX* genes should be considered only in a second step [37, 47, 80, 112, 131]. Extra-adrenal sympathetic paragangliomas (abdominal or thoracic) are more frequently caused by* SDHB*,* SDHD,* and* VHL* mutations [47, 77, 112]. Head and neck tumors are more frequently caused by* SDHD* (especially in presence of multiple tumors) and* SDHB* gene mutations and less often by* VHL* and* SDHC* gene mutations [44, 50, 96]. If patients are negative for mutations in these genes,* SDHAF2* might be considered for analysis [106]. Malignant tumors have been strongly associated (>30%) with germline mutations in* SDHB*, so initial analysis should address this gene [96]. If negative, then* VHL*,* NF1*,* SDHD,* or* MAX* genes can be considered for investigation [48, 96, 131]. It should be emphasized that mutations in the above-described genes may result in atypical phenotypes therefore rendering oriented genetic testing more complex.

A few and quick ways are likely to improve cost-effectiveness of molecular genetic testing. For instance, in VHL disease-associated pheochromocytoma, codon 167 of* VHL* gene appears as a first target, since missense mutations in this codon are strongly associated with pheochromocytoma development [60]. In addition, we should be aware of founder mutations already reported for* SDHx* genes in different countries such as the Netherlands, Poland, Italy, Spain, and Portugal in order to develop effective screening protocols [158–162].

Partial or large deletions may respond for false-negative results, when using conventional PCR followed by automatic sequencing techniques. Large deletions account for about 10% of the cases of* SDHx*-related paragangliomas [44]. Ideally, laboratories would routinely use methods for searching large genomic deletions such as quantitative multiplex PCR of short fluorescent fragments (QMPSF) or multiplex ligation-dependent probe amplification (MLPA) in order to minimize the risk of false-negative results.

Identification of a mutation allows tailoring treatment and follow-up therefore contributing to a better prognosis. The same holds true for the patients' relatives. In the specific cases of* RET*-associated pheochromocytoma, young relatives carriers of* RET* mutations may undergo prophylactic thyroidectomy to prevent the development of medullary thyroid carcinoma [67]. On the other hand, due to the higher risk of malignancy in patients carrying* SDHB* gene mutations, a closer biochemical and imaging follow-up might be provided in order to prevent the development of metastatic disease [95].

## 4. Conclusions and Future Perspectives

A great deal of knowledge has been added to the genetics of paragangliomas since the beginning of the millennium. Until then, the genes responsible for inheritable forms of paragangliomas were restricted to those underlying the syndromic forms of the disease;* RET* gene in multiple endocrine neoplasia type 2,* VHL* in von Hippel-Lindau disease; and* NF1* in neurofibromatosis type 1. The discovery of the succinate dehydrogenase genes associated with the development of familial paraganglioma syndromes, in particular the* SDHB* gene, frequently associated with malignant tumors, brought new insights into the management and prognosis of paragangliomas.

So far, at least 14 genes (*RET*,* VHL*,* NF1*,* SDHA*,* SDHB*,* SDHC, SDHD*,* SDHAF2*,* TMEM127*,* MAX*,* EGLN1*,* HIF2A*,* H-RAS,* and* KIF1B*) have been associated with the development of paragangliomas. These genes have been divided into two tumorigenesis clusters: a pseudohypoxic cluster (associated with mutations in* VHL*/*SDHx*/*EGLN1*/*HIF2A* genes) and a kinase receptor-signaling cluster (associated with* RET*/*NF1*/*TMEM127*/*MAX*/*KIF1B* gene mutations). Functional studies involving these genes and paraganglioma-associated mutations as well as gene expression profiles of tumor samples have greatly contributed to our understanding of tumorigenic pathways of paragangliomas. Progresses in genetic knowledge and the evidence for genotype-phenotype correlations have largely influenced the care of patients with positive impact.

In this review, we summarized the most relevant aspects regarding the genetics and clinical aspects of the syndromic and nonsyndromic forms of pheochromocytoma/paraganglioma aiming to provide an algorithm for genetic testing. Recent comprehension of the molecular pathways involved in the tumorigenesis of paragangliomas is likely to be improved by further functional assays, possibly hinting novel molecular-targeted therapy approaches.
